# Contour‐guided deep learning based deformable image registration for dose monitoring during CBCT‐guided radiotherapy of prostate cancer

**DOI:** 10.1002/acm2.13991

**Published:** 2023-05-25

**Authors:** Cédric Hemon, Bastien Rigaud, Anais Barateau, Florian Tilquin, Vincent Noblet, David Sarrut, Philippe Meyer, Julien Bert, Renaud De Crevoisier, Antoine Simon

**Affiliations:** ^1^ Univ Rennes, CLCC Eugène Marquis, INSERM, LTSI – UMR 1099 Rennes France; ^2^ Laboratoire des sciences de l'ingénieur de l'informatique et de l'imagerie ICube UMR 7357 Illkirch‐Graffenstaden France; ^3^ Université de Lyon CREATIS, CNRS UMR5220 Inserm U1294 INSA‐Lyon Université Lyon 1 Lyon France; ^4^ Department of Medical Physics Paul Strauss Center Strasbourg France; ^5^ Faculty of Medicine LaTIM, INSERM UMR 1101, IBRBS, Univ Brest Brest France

**Keywords:** CBCT, deformable registration, dose monitoring, prostate

## Abstract

**Purpose:**

To evaluate deep learning (DL)‐based deformable image registration (DIR) for dose accumulation during radiotherapy of prostate cancer patients.

**Methods and Materials:**

Data including 341 CBCTs (209 daily, 132 weekly) and 23 planning CTs from 23 patients was retrospectively analyzed. Anatomical deformation during treatment was estimated using free‐form deformation (FFD) method from Elastix and DL‐based VoxelMorph approaches. The VoxelMorph method was investigated using anatomical scans (VMorph_Sc) or label images (VMorph_Msk), or the combination of both (VMorph_Sc_Msk). Accumulated doses were compared with the planning dose.

**Results:**

The DSC ranges, averaged for prostate, rectum and bladder, were 0.60–0.71, 0.67–0.79, 0.93–0.98, and 0.89–0.96 for the FFD, VMorph_Sc, VMorph_Msk, and VMorph_Sc_Msk methods, respectively. When including both anatomical and label images, VoxelMorph estimated more complex deformations resulting in heterogeneous determinant of Jacobian and higher percentage of deformation vector field (DVF) folding (up to a mean value of 1.90% in the prostate). Large differences were observed between DL‐based methods regarding estimation of the accumulated dose, showing systematic overdosage and underdosage of the bladder and rectum, respectively. The difference between planned mean dose and accumulated mean dose with VMorph_Sc_Msk reached a median value of +6.3 Gy for the bladder and −5.1 Gy for the rectum.

**Conclusion:**

The estimation of the deformations using DL‐based approach is feasible for male pelvic anatomy but requires the inclusion of anatomical contours to improve organ correspondence. High variability in the estimation of the accumulated dose depending on the deformable strategy suggests further investigation of DL‐based techniques before clinical deployment.

## 1 INTRODUCTION

Large anatomical modifications occur during intensity modulated radiation therapy (IMRT) for prostate cancer which can increase the risk of target underdosage and toxicity.^1^, ^2^ Target displacements and organs at risk (OAR) deformations (e.g., volume changes) can be estimated using deformable image registration (DIR).^3^ DIR can be used between the planning computed tomography (CT) and fractionated daily anatomies, using onboard images such as cone‐beam CT, to estimate the accumulated dose for comparison to the planning dose. The accumulated dose is potentially important information for the clinical team to monitor the dose during the treatment and ensure that the target and OARs are receiving the prescription dose.^4^ Moreover, to account for large anatomical changes of the patient during treatment, one could use the accumulated doses to trigger replanning in order to correct any dose drift. Such strategy appears especially attractive in the context of online replanning which would enable the use of reduced PTV margins. Furthermore, the estimated accumulated dose could be useful for population analysis to improve organ specific toxicity models.^5^, ^6^

However, anatomical deformations estimation using DIR is challenging in the context of pelvic anatomy due to OARs content changes, treatment response, soft tissue interface (e.g., sliding), and poor contrast between soft tissue. Intensity‐based DIR, relying on dense deformation vector field (DVF) regularization, has been shown to be limited when estimating large deformations, especially in the context of poor contrast and lack of direct intensity correspondence.^7^ Contour‐guided DIR has been proposed to improve the estimation of soft tissue deformations leading to more reliable accumulated dose estimation.^8^, ^9^, ^10^ The uncertainties of this approach have been evaluated to be inferior to the difference between the planning and estimated delivered dose, using a finite element model (FEM) phantom and retrospective clinical data.^11^

Recently, deep‐learning (DL)‐based approaches have been proposed to solve automatization of time‐consuming radiation therapy planning processes such as anatomical structure segmentation,^12^, ^13^ pseudo CT estimation,^14^, ^15^ and dose planning.^16^ DL‐based DIR has been developed to estimate non‐rigid transformation between anatomical images with, however, remaining challenges to estimate large motion while providing similar or lower performance than conventional methods.^17^, ^18^ Using DL‐based registration methods, the exploitation of organs delineations thus needs to be evaluated in the context of fractionated radiation treatment to estimate accumulated doses before being deployed for clinical use.

This study proposes and evaluates a DL‐based DIR technique to estimate the accumulated dose during the course of radiotherapy for prostate cancer. The impact of the integration of organs delineations to guide the registration is especially addressed.

## 2 METHODS AND MATERIALS

### 2.1 Data

In this Institutional Review Board‐approved study, we retrospectively analyzed 364 images, including 23 planning computed tomography (CT) and 341 cone beam CT (CBCT) images, from 23 patients with localized prostate cancer.

All planning CT scans were acquired in a head‐first supine position with a rectal catheter and intravenous iodine contrast covering the sacroiliac joints to the lower edge of the lesser trochanters, using 3 mm slice thickness. The CBCT images were acquired in a head‐first supine position either weekly (17 patients corresponding to 132 images) or daily (6 patients corresponding to 209 images), during the 8 weeks of treatment. The CBCT images were acquired using either Varian On‐Board Imager (*n* = 16) or Elekta XVI (*n* = 7), with 1 mm slice thickness.

Philips Pinnacle® v9.4 treatment planning system (TPS) was used to generate a step‐and‐shoot IMRT planning dose distribution on the 23 patients planning CT scans. Organ delineation was set to meet GETUG (*Groupe d'Etude des Tumeurs Uro‐Génitales*) group recommendations.^19^ Rectum and bladder walls were derived from a 5 and 7 mm negative expansion from the manually delineated contours, respectively. The rectal length was defined as 1 cm below the planning target volume (PTV). Two clinical target volumes (CTVs) were delineated, CTV1 including the prostate and seminal vesicles and CTV2 including the prostate only. PTVs were generated from the CTVs by adding a 1 cm margin in all directions, except for the posterior direction, where 5 mm were added. The total dose was 46 Gy in PTV1, followed by a boost of 34 Gy to deliver 80 Gy in PTV2, at 2 Gy per fraction. Five 18 MV photon beams were used. GETUG dose–volume constraints were respected throughout: V70 Gy ⩽ 50% and Dmax ⩽ 80 Gy for the bladder wall, and V50 Gy ⩽ 50%, V72 Gy ⩽ 25%, and Dmax ⩽ 76 Gy for the rectum wall. Patients were asked to have a full bladder at the time of treatment. The CBCT images were also delineated, using the same recommendations.

### 2.2 Deep learning models

#### 2.2.1 Data preprocessing

The CBCTs were aligned on the CT anatomy using bone‐based rigid registration to account for patient positioning and to provide an initialization to the DIR process. For each patient, the CT and CBCT images were cropped to the smaller CBCT field of view and resized to 256*256*128 voxels using B‐Spline interpolation, corresponding to a resolution between 1.46 × 1.46 × 1.0 mm^3^ and 1.8 × 1.8 × 1.48 mm^3^.

#### 2.2.2 DIR with VoxelMorph

The VoxelMorph network^18^ was used to estimate the deformation between the planning CT and daily CBCT images for each patient. The original VoxelMorph is a weakly supervised U‐Net architecture that generates a deformation vector field (DVF) from the two inputs encoder/decoder. The regularization of the estimated DVF is performed by a diffusion regularizer on the spatial gradients leading to a smooth diffeomorphic transformation. The original VoxelMorph method can use manual segmentations to indirectly influence the training by computing the dice similarity coefficient (DSC) between the fixed and deformed moving input segmentations as a loss function. In this case, the segmentations are not exploited when inferring the deformation between new images, thus the network inputs are limited to the registered images. In this study, VoxelMorph has been extended to include anatomical structure segmentations (i.e., label images) on the fixed and moving images as inputs to extract segmentation specific features and contribute in the estimation of the DVF. After each iteration, the moving image segmentations were deformed according to the estimated DVF to compute a secondary loss function between the fixed and deformed label images.

In this study, a total of three VoxelMorph network variants were investigated: original VoxelMorph using either the anatomical or label images as inputs (VoxelMorph using Scans [VMorph_Sc] and VoxelMorph using Masks [VMorph_Msk]) and VoxelMorph with both scan and label images (4 inputs, VoxelMorph using Scans and Masks [VMorph_Sc_Msk]). For the latter, the original VoxelMorph method was modified to include delineations as inputs, in the channel dimension, and thus to exploit delineations not only for training, but also for inference. For the VoxelMorph networks including the manual segmentation as input (VMorph_Msk and VMorph_Sc_Msk), the label image included the values 0, 1, 2, 3, and 4 for the background, external body, rectum, bladder, and prostate masks, respectively. The fixed image was the CT, while the moving image was the CBCT.

**FIGURE 1:**
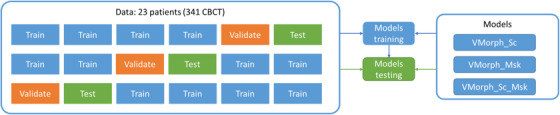
Three‐fold cross‐validation of the VoxelMorph models. Patients were randomly distributed in training, validation and test datasets.

The loss functions considered in the VoxelMorph models were:

(1) $$\begin{array}{ccc} L_{VMorph\_sc}\left(Scf,Scm,\phi \right) & = & \alpha_{1}NMI\left(Scf,Sc_{m}\circ \phi \right)\\ & & +\lambda_{1}L_{smooth}\left(\phi \right) \end{array}$$

(2) $$\begin{array}{ccc} L_{VMorph\_Msk}\left(Msk_{f},Msk_{m},\phi \right) & = & \alpha_{2}L1\left(Msk_{f},Msk_{m}\circ \phi \right)\\ & & +\lambda_{2}L_{smooth}\left(\phi \right) \end{array}$$

(3) $$\begin{array}{ccc} & & L_{VMorph\_Sc\_Msk}\left(Sc_{f},Sc_{m},Msk_{f},Msk_{m},\phi \right)\\ & & =\alpha_{3}NMI\left(Sc_{f},Sc_{m}\circ \phi \right)+\beta_{3}L1\left(Msk_{f},Msk_{m}\circ \phi \right) \end{array}$$

where $Sc_{f}$ (resp. $Msk_{f}$) and $Sc_{m}$ (resp. $Msk_{m}$) were the fixed and moving images (resp. fixed and moving masks) and ϕ was the deformation field. NMI(.) was the normalized mutual information,^20^ $L_{smooth}\left(.\right)$ the regularization function to encourage smooth deformation fields^18^ and L1(.) the L1 norm, that is, the sum of intensities absolute differences. The weighting parameters values, selected empirically, were: $\alpha_{1}=1,\lambda_{1}=4,\alpha_{2}=2,\lambda_{2}=4,\alpha_{3}=1,\beta_{3}=2\;and\;\lambda_{3}=4$ To train the VoxelMorph networks, the data was randomly separated into three‐folds of 70%, 15%, and 15% images that were successively used as training, validation, and test datasets (Figure 1). Random distribution of subjects was stratified based on number of CBCTs per patient and acquisiton system. Multiple images from individual patients were not distributed among the datasets. The image intensities were cropped around −1024 and 1575, and linearly normalized to the range [0, 1].

The VoxelMorph models were trained using the Adam optimizer with a learning rate scheduler starting at a value of 0.0005 and multiplied by 0.7 every 10 epochs. 150 epochs were used, and the epoch corresponding to the best evaluation measure in the validation set was chosen as the final model.

### 2.3 DIR with free‐form deformation

To compare the performance of the VoxelMorph methods with conventional methods, a free‐form deformation (FFD) based DIR method using the Elastix library^21^ was used as reference. The FFD was computed using the adaptive stochastic gradient descent optimizer and NMI loss function following a 4‐step multi‐resolution scheme where the image resolution was divided by factors of 4, 4, 2, and 1 with Gaussian smoothing. The spacing of the final grid was defined at 8.0 × 8.0 × 8.0 mm.

### 2.4 Evaluation and statistical analysis

Three sets of 3 VoxelMorph models were trained following the three‐folds of randomly distributed patients as training and test datasets (Figure 1). The statistical analysis was conducted on a total of 9 tests patients representing 6 patients with weekly images (*n* = 46) and 3 patients with daily images (*n* = 105). The CBCT images of six test patients were acquired with a Varian system, and three with an Elekta system.

Daily doses corresponding to CBCT images were estimated, considering dose deformation‐invariance,^22^ by rigidly aligning the planning dose on each CBCT image using the bone‐based rigid registration transformation resulting from the preprocessing step. For each patient, the DIR methods were used to estimate DVF between the planning CT and daily CBCT corresponding to the fixed and moving images, respectively. The resulting DVF were used to deform the CBCT images, doses, and anatomical contours (rectum, bladder, and prostrate) toward the planning anatomy. For each model and each patient, the deformed daily doses were summed on the planning anatomy to estimate the corresponding accumulated dose.

To quantitatively evaluate the performance of the DIR methods, geometric, and dosimetric metrics were computed for each patient. The geometric metrics corresponded to the DSC, average surface distance (ASD), Hausdorff Distance (HD), and 95th percentile of HD, between the planning and deformed daily contours, corresponding to the rectum, bladder, and prostate:

(4) $$DSC=2\frac{\left|A\cap B\right|}{\left|A\right|+\left|B\right|}$$

(5) $$\mathrm{ASD}=\frac{1}{N_{A}}\sum_{a\in S_{A}}\min_{b\in S_{B}}\left\|a-b\right\|$$

(6) $$\begin{array}{ccc} HD & = & \max\left(h\left(A,B\right),h\left(B,A\right)\right),\\ \mathrm{with}\;h\;\left(A,B\right) & = & \max_{{}_{a\in S_{A}}}\min_{b\in S_{B}}\left\|a-B\right\| \end{array}$$

where A and B correspond to the two considered segmentations (i.e., planning and deformed), |X| is the volume of the segmentation X, and S_X_ is the contour of the segmentation X. To assess the amplitude of deformations in the database, the DSC was also evaluated after the rigid registration.

The topology of the DVF estimated by each method was reported using the percentage of zeros and negative values of the determinant of the Jacobian inside each contour. Since no ground truth was available for accumulated dose, the impact of the different DIR methods was evaluated by a relative comparison with the planned dose. It corresponded to the report of the GETUG dose histogram volume (DVH) indices (D98% and V76Gy for the prostate, V70Gy for the bladder, and V72Gy for the rectum) for the planning doses and accumulated doses by each model.

The performances of the DIR approaches were compared using multivariate analysis of variance, using geometric and dosimetric metrics as dependent variables, followed by multiple analyses of variance (one for each of the metrics) and Tukey honestly significant difference (HSD) tests to determine pairwise differences in performance between models on each metric per organ. The confidence levels for each Tukey HSD test was considered significant if the *p* value was strictly inferior to 0.05.

Qualitative assessment of the DIR methods was conducted by reporting the deformed images, label, and dose with the corresponding DVF and determinant of Jacobian for the median case (i.e., patient) in terms of average DSC among the three anatomical structures.

## 3 RESULTS

The deep learning methods were implemented in Python 3.6 using Pytorch 1.10 with CUDA 10.2. The different models were trained and tested on an NVIDIA GTX 1080 Ti with 12 Go of VRAM. No overfitting was observed (no increase of the loss on the validation data).

### 3.1 Geometric evaluation

**FIGURE 2:**
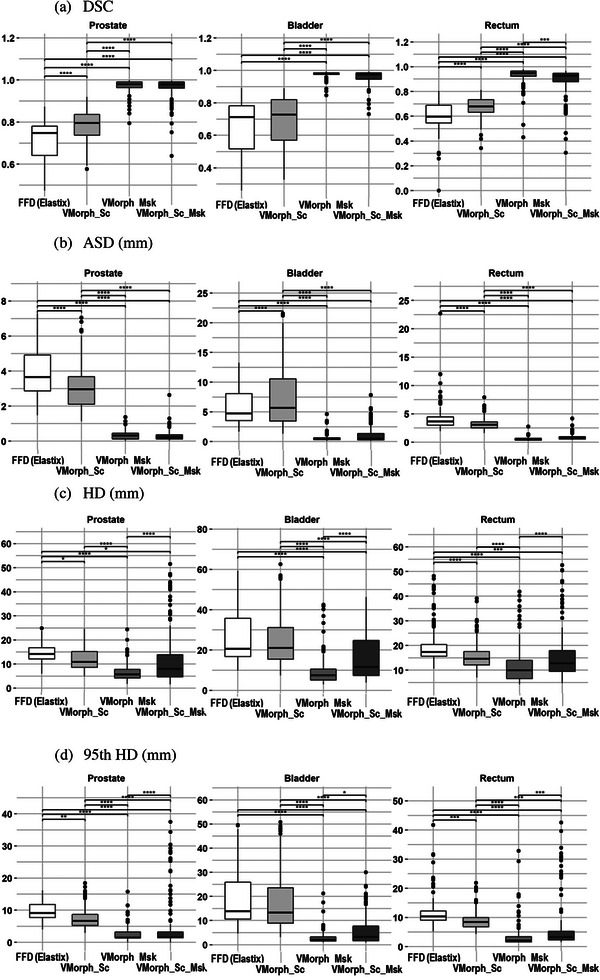
Geometric evaluation of the DIR methods for (a) DSC, (b) ASD, (c) HD, and (d) 95th HD metrics for the prostate, bladder, and rectum of the test dataset. *p < 0.05, **p < 0.01, ***p < 0.001, and ****p < 0.0001, adjusted analysis of variance with Tukey HSD test. Absence of * means difference was not statistically significant. DSC, dice similarity coefficient; HD, Hausdorff distance; MAD, mean absolute distance.

Figure 2a–d summarizes the DSC, DTA, HD, and 95th HD geometric metrics for the prostate, bladder, and rectum for the FFD and 3 VoxelMorph methods. The boxplots were computed using 148 values corresponding to the number of CBCTs of the 9 test patients. Results of the adjusted analysis of variance with Tukey HSD test are represented with *p < 0.05, **p < 0.01, ***p < 0.001, and ****p < 0.0001.

The DSC obtained after the rigid registration were (mean [min‐max]) 0.80 [0.58–0.96], 0.64 [0.29–0.90], 0.69 [0.31–0.82] for the prostate, bladder, and rectum, respectively.

Using only the intensity from the CT and CBCT images to drive the deformation, compared to the FFD method, the VoxelMorph_Sc method provided significantly better accuracy for prostate and rectum. Compared to the rigid registration, this method provided significantly better DSC only for the bladder.

When using the anatomical contours to drive the deformation, both VMorph_Msk and VMorph_Sc_Msk outperformed the rigid registration and intensity‐based deformable approaches. Comparing the VMorph_Msk and VMorph_Sc_Msk methods, the method using only the anatomical contours was significantly better for the rectum DSC and all HD metrics (*p* < 0.0001). In summary, the average DSC ranges were 0.60–0.71, 0.67–0.79, 0.93–0.98, and 0.89–0.96 for the FFD, VMorph_Sc, VMorph_Msk, and VMorph_Sc_Msk methods, respectively. The main discrepancies occurred when large differences appeared between planning and daily anatomies in terms of gas in the rectum.

Table 1 reports the percentage of voxels with a determinant of Jacobian inferior or equal to zero for each DIR method and organ. In summary, the average percentage ranges were 0.01–0.07, 0–0, 0–0.05, and 0.76–1.90 for the FFD, VMorph_Sc, VMorph_Msk, and VMorph_Sc_Msk methods. Compared to other methods, the VMorph_Sc_Msk had significantly higher percentage of deformation causing image folding. Qualitatively, this folding was mainly located near some masks boundaries, which may be explained by a conflict between the contributions of the masks and the intensities of the scans.

**TABLE 1:** Percentage of voxels with a determinant of Jacobian inferior or equal to zero per DIR method for the prostate, bladder, and rectum.

	Mean (min–max) percentage of determinant of Jacobian ≤0		
Methods	Prostate	Bladder	Rectum
**FFD (Elastix)**	0.01 (0–0.80)	0.07 (0–4.96)	0.02 (0–1.92)
**VMorph_Sc**	0 (0–0)	0 (0–0)	0 (0–0)
**VMorph_Msk**	0.05 (0–0.46)	0.01 (0 ‐ 0.23)	0 (0–0.01)
**VMorph_Sc_Msk**	1.90 (0.19–6.18)	1.31 (0.12–4.37)	0.76 (0–3.25)

**FIGURE 3:**
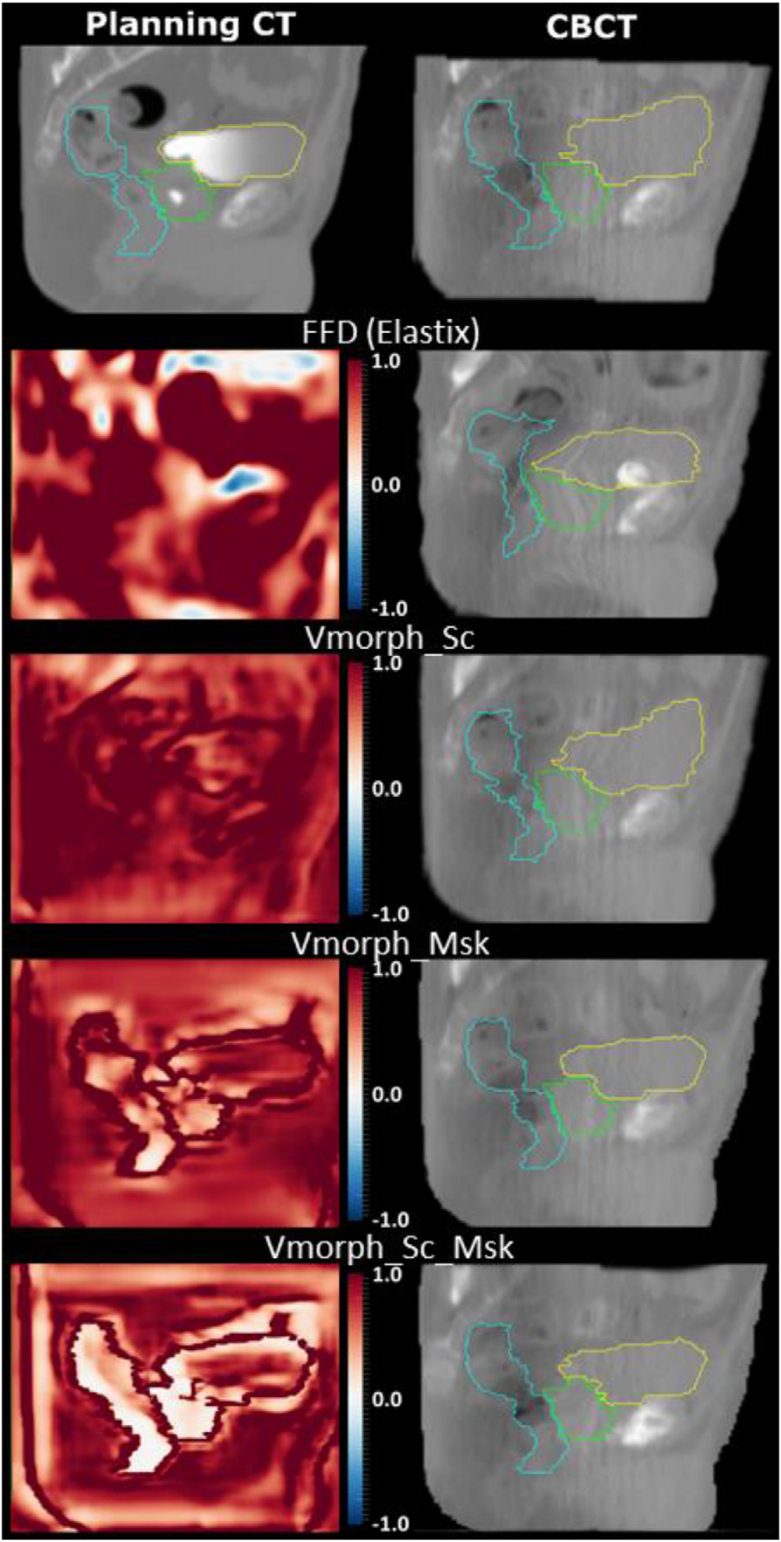
Qualitative evaluation of deformations per method for a median case in terms of DSC. The first row contains the planning and CBCT images. The second to fourth rows contain the Jacobian determinant and deformed CBCT images. The green, yellow, and light blue colors represent the prostate, bladder, and rectum, respectively. The image intensity parameters were 2000 and 0 for the window and level, respectively. The determinant of Jacobian intensity parameters were 2 and 0 for the window and level, respectively.

Figure 3 shows the planning CT, original CBCT, and the deformed CBCTs with their corresponding determinant of Jacobian images for each method for a patient corresponding to the median DSC.

### 3.2 Relative dosimetric evaluation

**FIGURE 4:**
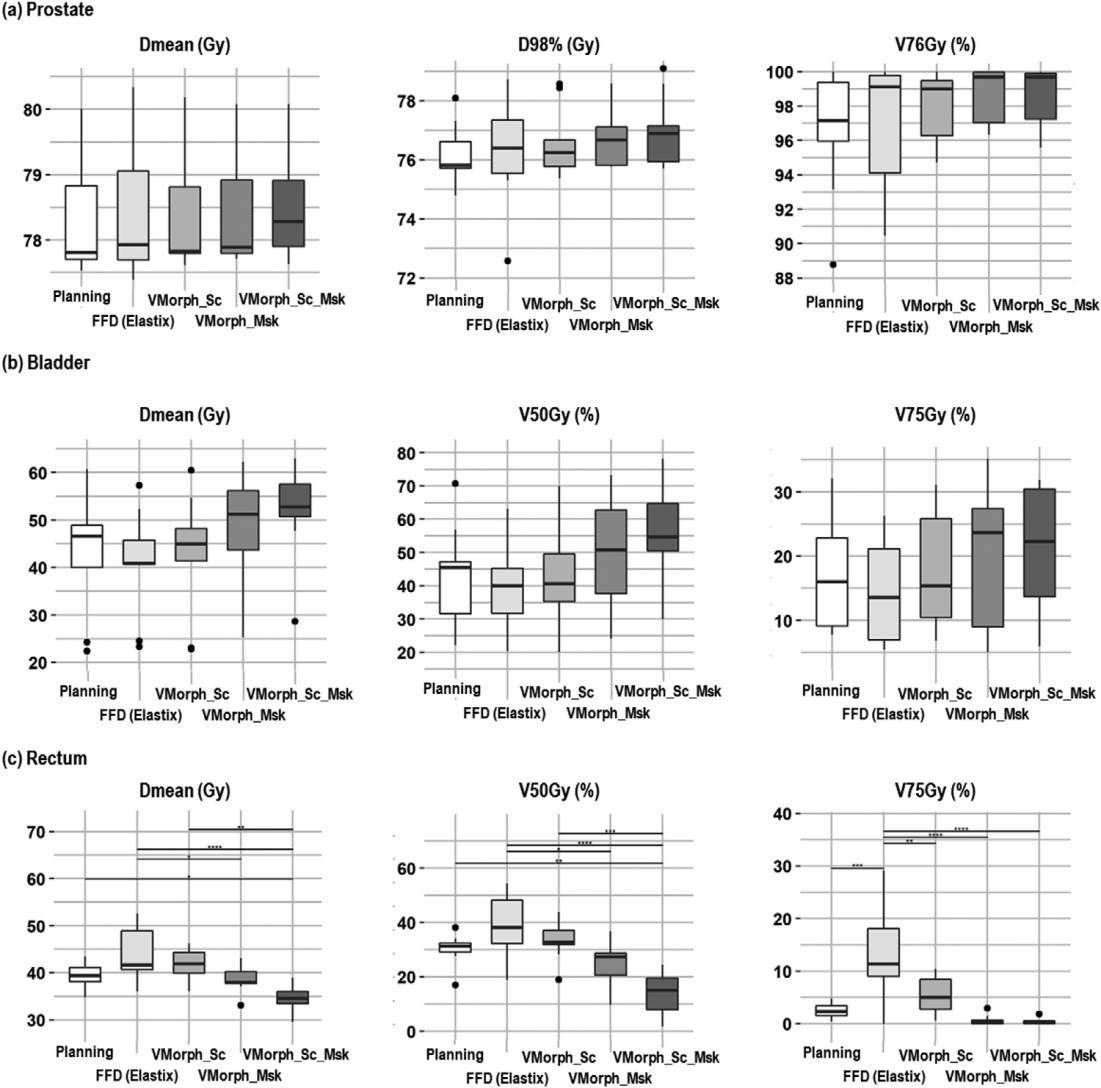
Dosimetric evaluation of accumulated doses per DIR method compared to the planning dose for the prostate, bladder, and rectum of the test dataset. *p < 0.05, **p < 0.01, ***p < 0.001, and ****p < 0.0001, adjusted analysis of variance with Tukey HSD test. Absence of * means difference was not statistically significant.

**FIGURE 5:**
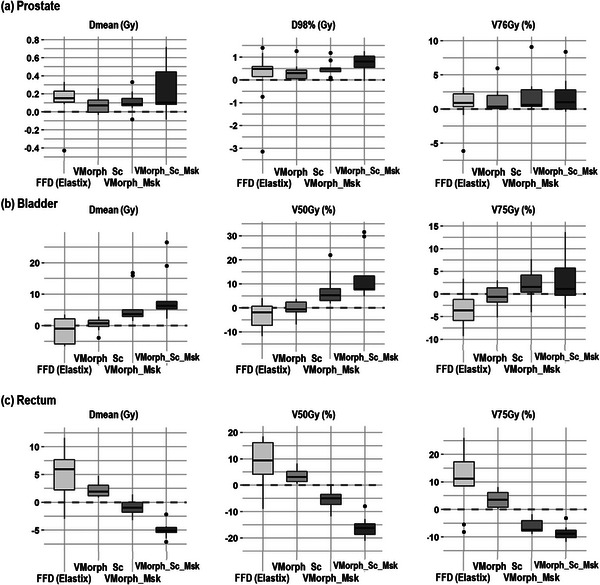
Dosimetric evaluation of accumulated dose differences with the planning dose per DIR method for the prostate, bladder, and rectum of the test dataset.

Figure 4 reports the DVH indices of each organ for the accumulated doses per DIR method compared to the planning dose on the test dataset. Figure 5 reports the difference of the DVH indices between the planning and accumulated doses. For the prostate, no significant differences were observed between the estimated accumulated doses by the DIR methods compared to the planned dose. For the bladder, while being not significant, the VoxelMorph approaches estimated increased accumulated doses compared to the planning. For the rectum, the VoxelMorph methods including the contours systematically estimated significantly decreased accumulated doses.

**FIGURE 6:**
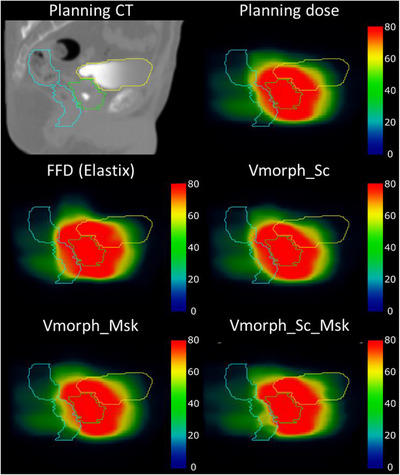
Qualitative evaluation of accumulated doses per DIR method for the overall median case regarding DSC. The first row contains the planning CT and dose. The second and third rows contain the accumulated dose per method with the planning contours. The green, yellow, and light blue colors represent the prostate, bladder, and rectum, respectively. The dose intensity parameters were 80 and 40 in Gy for the window and level, respectively.

Figure 6 shows the planning image, planning dose, and accumulated doses for each method for the patient corresponding to the median DSC.

## 4 DISCUSSION

This study evaluated the geometric and dosimetric performances of the FFD and DL‐based DIR methods to estimate anatomical deformation during prostate radiotherapy for the estimation of the delivered dose. Our results suggest that the DL‐based method combining the image and the delineations information is able to register the male pelvic anatomy between the planning CT and daily CBCT and is therefore a good candidate for dose monitoring during the course of radiotherapy. The inclusion of the contours resulted in an improved anatomical correspondence, in regard to these contours. Indeed, only the two methods including the delineations were able to meet the tolerance values proposed in TG132^23^ (i.e., DSC > 0.8 and MAD < 3 mm), except for a limited number of CBCT images. For example, the VoxelMorph method combining contours and original images (VMorph_SC_Msk) provided DSC below 0.8 for the prostate and bladder on 3 CBCT images (i.e., 2.0% of the tested images), and for the rectum on 10 CBCT images (i.e., 6.6% of the tested images). These lower performances were mainly related to very large differences in terms of rectum gas. If combining contours with original images resulted to a significantly higher percentage of image folding, the exploitation of masks only may result to unrealistic deformation outside the masks, since no information is provided to guide the registration at these positions, as illustrated in Figure 7 (Supplementary materials). The DL‐based methods always outperformed the FFD geometric performance while being faster to compute during inference (seconds vs. minutes).

Comparing the accumulated doses between each method, our results highlight that large discrepancies potentially exist in the estimation of the received dose during prostate treatment. Especially, large differences were present in the bladder and rectum between the two VoxelMorph methods including the anatomical contours in the inputs while providing similar geometric metric accuracies. This indicates that small differences impacting the behavior of the DL‐based algorithm can have large impact on the estimated deformations which could not be captured by standard geometric metrics. It also confirms that different DIR methods may result to large differences in terms of accumulated doses.^8^, ^24^ In that context, further analysis needs to be conducted to locally evaluate the robustness of DL‐based approaches, especially with complex dataset, an independent external cohort or numerical phantoms.^25^ While the VoxelMorph method includes an iterative diffeomorphic regularization during training, the approach using both anatomical and label images estimated more DVF folding (up to 6% in the prostate). This could be explained by the necessity of satisfying two loss functions, with one being the NMI between two images with no direct intensity correspondence (e.g. Hounsfield unit, contrast agent, tissue disappearance, gas pocket). This resulted in more complex and heterogeneous deformations with high deformation gradient at the border of the organs while providing more homogeneous determinant of Jacobian inside (Figure 3). Future DL‐based strategies could integrate a determinant of Jacobian penalty to improve DVF inversibility. Moreover, while the proposed methods exploit delineations as inputs, this approach may be improved using multi‐tasking learning strategies to couple the tasks of automatic segmentation and contour propagation via DVF estimation, improving both results using prior patient specific information.^26^, ^27^

To our knowledge, no other study investigated the usage of DL‐based DIR to perform pelvic dose accumulation during prostate radiotherapy. However, previous studies investigated the variability in estimating the delivered dose. Using weekly CTs of 9 prostate patients, one study reported DIR‐based bladder DSC ranges of 0.80–0.94 and observed large Dmean variation between the estimation methods (e.g., static DVH addition, intensity‐based DIR, and contour‐based DIR) of the bladder with difference up to 11 Gy between the DIR‐based methods.^28^ In comparison, we observed in our study an average DSC of the bladder ranging between 0.65 and 0.98 with the accumulated Dmean variation ranging between −1.54 and 9.44 Gy compared to the planning dose (Figure 5). For the rectum, the trends were inverted with average DSC ranging between 0.60 and 0.93 and Dmean variation ranging between 4.68 to −5 Gy (Figure 5). Recently, one study investigated the difference between the planning and accumulated doses of 338 CBCTs from 23 patients using the ANACONDA^10^ DIR method.^29^ Comparing the planning and accumulated doses, this study observed no significant difference for the Dmean of the rectum and significant higher difference (+0.6 Gy in average) for the Dmean of the bladder. The variations in estimating the accumulated doses observed in the literature and in our study suggest that DIR‐based clinical decisions need to be carefully approved, preferably with qualitative evaluation from trained clinicians.

Our study has several limitations. First, a cross validation training strategy was used to compensate for the low number of patients which can overestimate the performance of the trained DL‐models. The VoxelMorph model needs to be further evaluated on a large cohort with an external dataset to investigate its robustness. Second, the geometric evaluation was computed on the same anatomical contours that were used as input to train the model, and the CBCT delineations exploited in this work were obtained manually. However, DL‐based automatic segmentation models are becoming popular and are now being implemented in commercially available TPSs and should be accessible to be integrated in contour‐driven DIR‐based methods. A similar study could be performed using MR images or another anatomical localization (e.g., head and neck), especially important for dose accumulation estimation in the context of MRLINAC treatment. Third, daily dose distributions were estimated by rigidly aligning planning dose distributions on CBCT images. CBCT dose calculation methods, for example, based on Hounsfield Units override or synthetic CT generation, would provide more accurate dose distributions.^30^ Finally, the dosimetric evaluation was limited to a relative comparison to planned dose, since no ground truth was available for accumulated dose. To generate such a reference, the use of well‐defined anatomical landmarks, which is challenging when considering soft tissues and CBCT images, should be investigated.

## 5 CONCLUSION

The estimation of the deformations using DL‐based approach is feasible for male pelvic anatomy but requires the inclusion of anatomical contours to improve organ correspondence in context of multimodality, contrast agent, and medical device inserted. High variability in the estimation of the accumulated dose depending on the deformable strategy suggests further investigation of DL‐based techniques before clinical deployment.

## AUTHOR CONTRIBUTIONS

Antoine Simon and Renaud de Crevoisier were involved with the initiation and design of the study, and data management. Antoine Simon and Vincent Noblet participated to the method selection and design. Cédric Hémon and Florian Tilquin proposed and implemented the methods. Bastien Rigaud performed the data analysis and manuscript writing. Anaïs barateau performed the dose calculations. David Sarrut, Philippe Meyer, and Julien Bert provided expertise in radiation therapy data management and image processing methods design. All authors were involved in reviewing and approving the manuscript.

## CONFLICT OF INTEREST STATEMENT

The authors declare no conflicts of interest.

## Supporting information

Supporting Information
