# Supplementary material for: Contour‐guided deep learning based deformable image registration for dose monitoring during CBCT‐guided radiotherapy of prostate cancer
Source: J Appl Clin Med Phys. 2023 May 25;24(8):e13991. doi: 10.1002/acm2.13991 (PMC10445205; doi:10.1002/acm2.13991)
Supplement: Supplementary file 1 — Supporting Information [file ACM2-24-e13991-s001.docx]

**Fig. 7 - supplementary.** Example of deformed CBCT images obtained with methods VMorph_Msk (a) and VMorph_Sc_Msk (c). The corresponding reference CT image is represented in (b). In this case, the method VMorph_Msk results to unrealistic deformations (deformations of the bones), while this is not observed with the VMorph_Sc_Msk method.


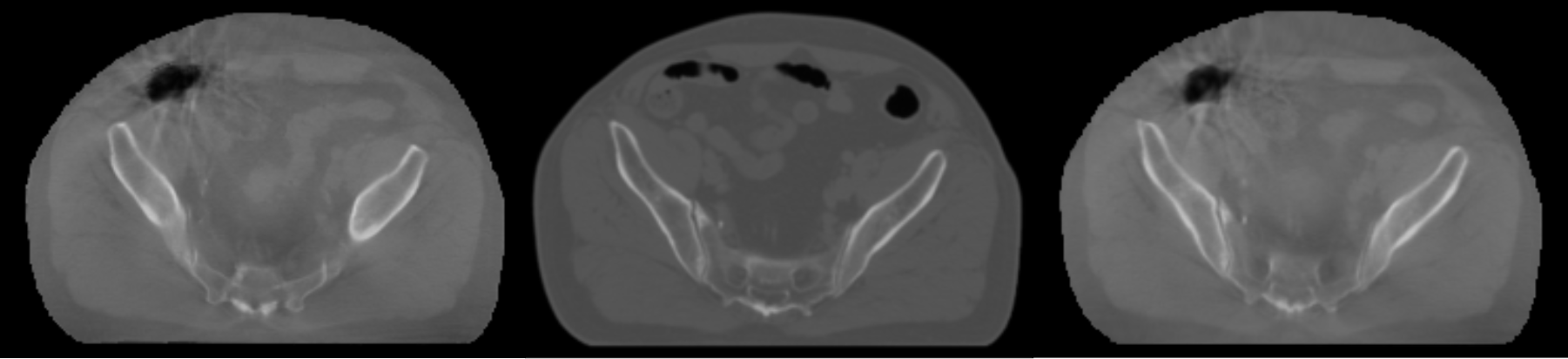


(a) CBCT deformed by VMorph_Msk (b) Reference CT image (c) CBCT deformed by VMorph_Sc_Msk
